# Morphology, Distribution and Phenotype of Polycystin Kidney Disease 2-like 1-Positive Cerebrospinal Fluid Contacting Neurons in The Brainstem of Adult Mice

**DOI:** 10.1371/journal.pone.0087748

**Published:** 2014-02-04

**Authors:** Adeline Orts-Del’Immagine, Anne Kastner, Vanessa Tillement, Catherine Tardivel, Jérôme Trouslard, Nicolas Wanaverbecq

**Affiliations:** Aix-Marseille Université (AMU), Laboratoire de Physiologie et Physiopathologie du Système Nerveux Somato-moteur et Neurovégétatif (PPSN) - EA 4674, Faculté des Sciences St. Jérôme, Marseille, France; Institut Curie, France

## Abstract

The mammalian spinal cord and *medulla oblongata* harbor unique neurons that remain in contact with the cerebrospinal fluid (CSF-cNs). These neurons were shown recently to express a polycystin member of the TRP channels family (PKD2L1) that potentially acts as a chemo- or mechanoreceptor. Recent studies carried out in young rodents indicate that spinal CSF-cNs express immature neuronal markers that appear to persist even in adult cells. Nevertheless, little is known about the phenotype and morphological properties of medullar CSF-cNs. Using immunohistochemistry and confocal microscopy techniques on tissues obtained from three-month old PKD2L1:EGFP transgenic mice, we analyzed the morphology, distribution, localization and phenotype of PKD2L1^+^ CSF-cNs around the brainstem and cervical spinal cord central canal. We show that PKD2L1^+^ CSF-cNs are GABAergic neurons with a subependymal localization, projecting a dendrite towards the central canal and an axon-like process running through the parenchyma. These neurons display a primary cilium on the soma and the dendritic process appears to bear ciliary-like structures in contact with the CSF. PKD2L1^+^ CSF-cNs present a conserved morphology along the length of the medullospinal central canal with a change in their density, localization and dendritic length according to the rostro-caudal axis. At adult stages, PKD2L1^+^ medullar CSF-cNs appear to remain in an intermediate state of maturation since they still exhibit characteristics of neuronal immaturity (DCX positive, neurofilament 160 kDa negative) along with the expression of a marker representative of neuronal maturation (NeuN). In addition, PKD2L1^+^ CSF-cNs express Nkx6.1, a homeodomain protein that enables the differentiation of ventral progenitors into somatic motoneurons and interneurons. The present study provides valuable information on the cellular properties of this peculiar neuronal population that will be crucial for understanding the physiological role of CSF-cNs in mammals and their link with the stem cells contained in the region surrounding the medullospinal central canal.

## Introduction

Medullospinal cerebrospinal fluid contacting neurons (CSF-cNs) are part of the circumventricular organs found in the central nervous system (CNS). Some of these organs form a bridge between the CSF, the bloodstream, and neurons in the parenchyma and are also believed to be involved in neuronal volume transmission [1]–[3]. These cells are present in the wall of the ventricular cavities and around the cc and, depending on the position of their soma, they represent intra-, subependymal and distal CSF-cNs. The network of the medullospinal CSF-cNs might represent the simplest circumventricular organ and are dispersed along the entire length of the central canal (cc) of the spinal cord from its terminal filum at the caudal level to the *medulla oblongata* at more rostral levels [3], [4]. CSF-cNs are present in all chordates from the lancelet to the human but despite this strong evolutionary conservation and morphological descriptions in numerous species, their functions remain largely unknown [4], [5].

In mammals, most of our knowledge concerning the medullospinal CSF-cN system comes from studies conducted at the spinal cord level [6]–[11]. These cells have a soma, more or less inserted in the ependymal layer lining the cc, from which emerges an extension terminated by a typical bulb-like structure (bud) that comes into contact with the CSF [6]–[12]. Depending on the species, this bud carries a sensory primary cilium, a kinocilium or stereocilia [4], [5]. The presence of these organelles has led to the accepted view that CSF-cNs may have sensory functions. Specifically, these neurons might sense modifications in the composition of surrounding CSF (chemical hypothesis) or in the pressure/flow of CSF (mechanical hypothesis) [4], [5]. They were also suggested to be in contact with the Reissner fiber that originates from the subcommisural organ and extends to the terminal filum [4], [5].

The chemical hypothesis is supported by the presence of various bioactive molecules such as hormones and neurotransmitters circulating in the CSF that are thought to come in contact with CSF-cNs [2], [13]–[15]. In addition, spinal CSF-cNs have been shown to express ionic channels of the polycystin (TRPP or polycystin kidney disease 2-like 1, PKD2L1) transient receptor potential subtype; these channels are sensitive to extracellular pH and osmolarity variation [12], [16]. We showed in a previous study [12] that PKD2L1^+^ CSF-cNs are also present in the dorsal vagal complex (DVC), a hindbrain region dedicated to the regulation of important autonomic functions [17], [18]. Our work indicated that CSF-cNs close to the cc are PKD2L1^+^ and sensitive to changes in pH and osmolarity of the extracellular fluid. These neurons displayed robust action potential discharge activity as well as GABA and glycinergic synaptic currents. However, their phenotype and their degree of maturity have not yet been determined.

A renewed interest in these particular neurons emerged from the demonstration that the region surrounding the spinal cc, where CSF-cNs are found, represents a stem cell niche and may generate both neurons and/or glial cells in lower vertebrates [19], [20] as well as in mammals [21], [22]. Within this spinal niche, however neurogenesis appeared very limited or inexistent in adult mammals [23]–[26]. Interestingly, the DVC, where we previously reported the presence of CSF-cNs, has also been shown to contain neural stem cells [27], [28]. CSF-cNs are generated during embryogenesis, and have been shown to remain in a “stand-by mode” in young rodents: they display a certain degree of immaturity in terms of excitability [8], [11] and still express several markers of young immature neurons such as growth associated protein 43 (GAP43) [11], doublecortin (DCX), polysialylated-neural cell adhesion molecule (PSA-NCAM), as well as HuC/D [8], [11]. Recent studies have suggested that some spinal CSF-cNs retain expression of such markers even in adults and that this “stand-by mode” might be conserved at later stages [8], [29]. In addition, in both young and young-adult rats, CSF-cNs were not found to express the neuronal nuclei protein (NeuN) [8], a marker of neuronal maturity [30].

Our first aim was therefore to carry out an in-depth analysis of the morphology, distribution and localization of PKD2L1^+^ CSF-cNs in the cervical spinal cord and brainstem of adult mice. Secondly, we sought to quantitatively characterize the degree of cellular maturity of these cells by measuring markers of immature and mature neurons, and by doing so to additionally compare the phenotype of medullar S-CSF-cNs to that of cervical spinal CSF-cNs.

We show that S-CSF-cNs are present, with a conserved morphology, along the entire length of the medullospinal cc and bear a primary cilium on the soma, not on the bud. These cells are mainly GABAergic and strongly express PKD2L1 proteins on the somatodendritic compartment. Our findings confirm that this channel represents a specific marker for medullospinal CSF-cNs. In addition, we demonstrate that, at adult stages, CSF-cNs remain in an intermediate stage of maturity, as they continue to express DCX, Hu/CD and Nkx6.1 in the presence of NeuN.

## Materials and Methods

### Ethics Statement

This study was carried out in strict conformity with the recommendations and rules set by the EC Council Directive (2010/63/UE) and the French “Direction Départementale de la Protection des Populations des Bouches-du-Rhône”. Prior to any experimental procedure, animals were anaesthetized using a mixture of Xylazine/Ketamine and euthanized (see below). All efforts were made to insure animal well-being and minimize animal suffering and the number of animals used. The experimental procedures have been approved by our local Animal Care Ethical Committee (Comité Ethique de Provence N°14) (licence N°13.435 held by JT and N°13.430 by NW). Animals were housed at constant temperature (21°C) under a standard 12 h light-12 h dark cycle, with food (pellet AO4, UAR, Villemoisson-sur-Orge, France) and water provided *ad libitum.*

### Animals

We used PKD2L1:EGFP positive and negative transgenic mice obtained by crossing PKD2L1-IRES-Cre with Z/EG reporter transgenic mice. Thus, in PKD2L1^+^ cells, EGFP expression was selectively induced by CRE recombinase activity [16]. PKD2L1-IRES-Cre mice were a generous gift from Dr CS Zuker (Howard Hughes Medical Institute, University of California, La Jolla, USA) and Z/EG reporter lines were kindly provided by Dr P Durbec (IBDML, Aix-Marseille Université, France). To assess EGFP expression in PKD2L1^+^ animals, we carried out PCR on tail genomic DNA. CRE transgene was detected using the forward primer 5′-CGT ACT GAC GGT GGG AGA AT-3′ and the reverse primer 5′-CCC GGC AAA ACA GGT AGT TA-3′, with the following PCR conditions: 7 min at 95°C, 35 cycles at 95°C for 40 sec, 62°C for 40 sec and 72°C for 40 sec, followed by a final step at 72°C for 6 min. The amplicon size was 166 bp. EGFP was detected using the forward primer 5′-GCC ACA AGT TCA GCG TGT CC-3′ and the reverse primer 5′-GCT TCT CGT TGG GGT CTT TGC-3′, with the following PCR conditions: 5 min at 95°C, 36 cycles at 95°C for 30 sec, 64°C for 30 sec and 72°C for 40 sec, followed by a final step at 72°C for 7 min. The amplicon size was 573 bp.

### Immunohistochemistry

Three-month-old adult mice were anaesthetized with an intraperitoneal injection of ketamine (Carros, France) and xylazine (Puteau, France) mixture (100 and 15 mg/kg, respectively) and transcardially perfused with phosphate buffer solution (PBS at 0.1 M). Subsequently the animals were perfused with 4% PFA in PBS. For the experiments where GABA immunoreactivity was tested, PKD2L1:EGFP positive animals were transcardially perfused with a fixation medium containing 4% PFA and 0.2% glutaraldehyde. Brains and spinal cords were immediately removed, post-fixed one hour in 4% PFA at 4°C, rinsed in PBS (at 4°C overnight, ON), cryoprotected for 24 to 48 hours in 30% sucrose at 4°C and frozen in isopentane (−40°C).

**Figure 1:**
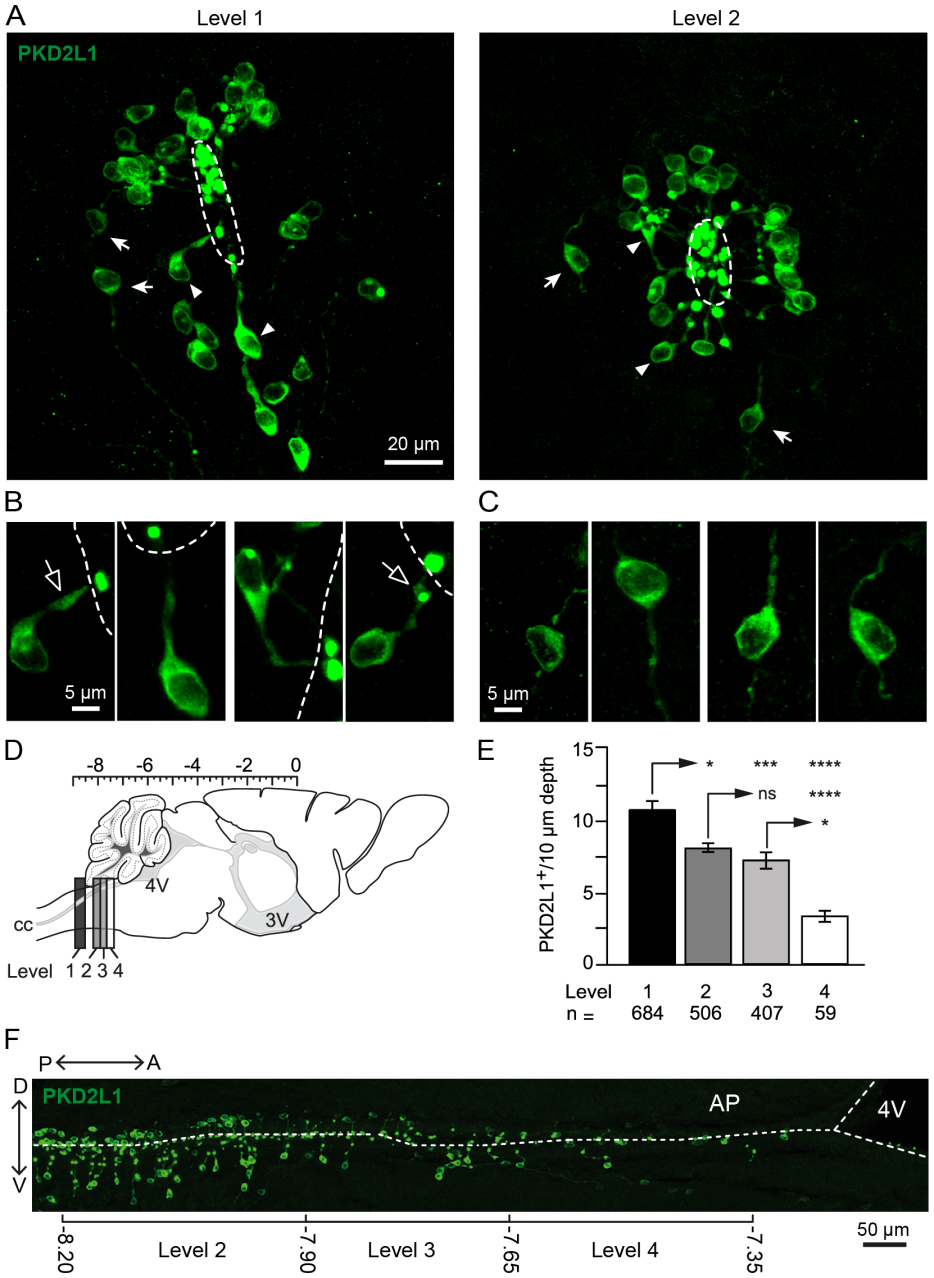
PKD2L1^+^ S-CSF-cNs are present with a different density along the central canal. A. Representative coronal sections at level 1 (Left) and level 2 (Right) showing the presence of PKD2L1^+^ cells sitting outside the ependymal layer around the cc. The majority of the cells immunolabeled with antibodies against PKD2L1 exhibited the classical morphology of CSF-cNs with a thick projection ended by a bud (arrowheads). Fewer PKD2L1^+^ cells had a bipolar morphology, thin processes and did not contact the cc (arrows). Examples, at a higher magnification, of PKD2L1^+^ cells that contacted (**B**) or did not contact (**C**) the cc. Note that cells from the latter type were excluded from our analysis. **D.** Sagittal scheme of the mouse brain and cervical spinal cord showing the regions selected for the morphological and phenotypical analysis from level 1 to 4 (dark to white boxes). Scale on top: stereotaxic coordinates in the antero-posterior axis from Bregma (0 mm; Paxinos Mouse Brain Atlas). **E.** Summary bar graph of the average number of PKD2L1^+^ CSF-cNs present per 10 µm of tissue depth at levels 1 to 4. ns: not significant; *, P<0.05; ***, P<0.001 and ****P<0.0001. Level number and the corresponding analyzed cells number (n) are indicated below the graph. **F.** Representative sagittal section of the medullospinal region showing the distribution of PKD2L1^+^ cells around the cc from the lower brainstem to the 4V. The brackets below the image delimit the 3 levels of the brainstem (from 2 to 4) analyzed in the present study with the mention of the stereotaxic coordinates (in mm). Double arrows: left and top for dorsal-ventral (V-D) and postero-anterior (P-A) orientation of the section, respectively. Central canal (cc) and 4^th^ ventricle (4V) are delineated with white dash lines.

Brainstem and cervical spinal cord coronal or sagittal thin sections (40 µm) were obtained using a cryostat (Leika CM3050) and collected serially in twelve-well plates containing 0.1 M PBS. In the analysis carried out in our study, we distinguished the cervical spinal cord region (level 1, antero-posterior stereotaxic coordinates of regions more caudal than −8.50 mm from Bregma; Paxinos Mouse Atlas and see fig. 1D) and three regions for the brainstem: level 2 (from −8.20 to −7.90 mm), level 3 (from −7.90 to −7.65 mm) and level 4 (from −7.65 to −7.35 mm). Following one hour incubation in the blocking solution with a composition optimized for a specific labeling experiment (see Table 1), sections were incubated ON or for 48 hours at 4°C with the primary antibody (see description in Table 1). Typically in double immunohistochemical labeling experiments, the two primary antibodies (see Table 1) were applied sequentially. Sections were then washed in PBS and incubated for two hours with the secondary antibody conjugated either to AlexaFluor 488 or 594 (1∶400; all secondary antibodies were purchased from Life Technologies). To determine whether S-CSF-cNs were cholinergic, we labeled brainstem sections obtained from choline acetyltransferase (ChAT)-EGFP adult mice (gift from Dr M Cordero-Erausquin, INCI, Strasbourg, France) with a primary antibody against PKDL21. After several washes in PBS, sections were mounted on 4% gelatin coated slides and coverslipped with Mowiol mounting medium for fluorescence microscope preparation. To asses for the selectively of the observed immunolabeling, in each set of experiments, sections were treated in the absence of primary antibodies (negative control) and regions known to express the analyzed marker were visualized (positive control). Typically, experiments were performed on tissues obtained from three different 3-month-old animals and each set conducted on non consecutive sections for each level.

**Table 1:** List of primary, secondary antibodies and experimental procedures used to label medullospinal CSF-cNs.

Antigen	Host	Primary antibodies/Dilution	Serum (in PBS)	Distributor Cat. Nr.	Incubation at 4°C	Secondary antibodies
**Ac-αTub**	Mouse	Monoclonal 1∶400	Triton (0.3%),BSA (4%), NGS (2%)	Sigma T6793	ON	Goat anti mouse 594
**AC3**	Rabbit	Polyclonal 1∶500	Triton (0.3%),BSA (1%), NGS (3%)	Santa Cruz sc-588	ON	Goat anti rabbit 594
**DCX**	Goat	Polyclonal 1∶100	Triton (0.1%),HS (5%)	Santa Cruz sc-8066	48 h	Donkey anti goat 594
**GABA**	Rabbit	Polyclonal 1∶250	Triton (0.3%),HS (5%)	Immunostar 20094	48 h	Goat anti rabbit 594
**GAD67**	Mouse	Monoclonal 1∶500	NGS (3%)	Millipore MAB5406	48 h	Goat anti mouse 594
**GFP**	Mouse	Monoclonal 1∶500	Triton (0.1%),HS (3%)	ABCAM ab38689	48 h	Goat anti mouse 488
	Rabbit	Polyclonal 1∶7500	Triton (0.05%),HS (5%)	* Gift from Dr. T DOAN	ON	Goat anti rabbit 488
**HuC/D**	Mouse	Monoclonal 1∶1000	Triton (0.1%),NGS (5%)	Molecular Probes A-21271	ON	Goat anti mouse 594
**MAP2**	Mouse	Monoclonal 1∶600	Triton (0.3%),HS (3%), BSA (1%)	Sigma M-1406	48 h	Goat anti mouse 594
**NeuN**	Mouse	Monoclonal 1∶800	Triton (0.3%),BSA (4%), NGS (2%)	Millipore MAB377	ON	Goat anti mouse 594
**NF-160**	Mouse	Monoclonal 1∶80	Triton (0.3%),HS (3%), BSA (1%)	Sigma N5264	ON	Goat anti mouse 594
**Nkx6.1**	Mouse	Monoclonal 1∶100	Triton (0.1%),BSA (1%), NGS (3%)	^#^ Dev.Stud. Hybrid.Bank F55A10	ON	Goat anti mouse 594
**PSA-NCAM**	Mouse IgM	Monoclonal 1∶250	Triton (0.3%),BSA (1%), HS (3%)	Millipore MAB5324	ON	Goat anti mouse IgM 594
**PKD2L1**	Rabbit	Polyclonal 1∶700	Triton (0.3%),BSA (1%), HS (3%)	Millipore AB9084	ON	Donkey anti-rabbit 488or Goat anti rabbit 488
**TH**	Mouse	Monoclonal 1∶1000	Triton (0.3%),NGS (3%)	Millipore MAB318	ON	Goat anti mouse 594
**5-HT**	Goat	Polyclonal 1∶400	Triton (0.3%),HS (5%)	Immunostar 20079	ON	Donkey anti goat 594

List of the primary antibodies with the experimental conditions (dilution, blocking medium and incubation time) for the different sets of experiment carried out to label S-scNs. The supplier name and catalog number is also mentioned along with the nature of the secondary antibody and associated fluorochrome.

* Rabbit anti-GFP antibody purified as described by Rudner and Losick [66] is a gift from Dr T Doan (Laboratoire de Chimie Bactérienne – CNRS, Marseille, France) [66]. ^#^ Nkx6.1 antibody is a generous gift from Dr JP Hugnot, (INM, Montpellier, France) and was obtained from the Developmental Studies Hybridoma Bank (Dev. Stud. Hybrid. Bank, University of Iowa).

### Image Acquisition

Sections were observed on a confocal laser scanning microscope (CLSM; Zeiss LSM700) equipped with solid state fiber optic lasers and single plane images or stacks of images were acquired with a 2x digital zoom using either a 20x objective (numerical aperture, NA: 0.8 for an optical thickness of ∼2 µm) or a 63x oil immersion objective (NA: 1.4 for an optical thickness of ∼0.8 µm). Images have been acquired at optimal resolution between 512×512 and 1024×1024 pixels. To insure the continuity of the image acquisition, each plane acquired for a stack overlapped with the next plane. When using two fluorochromes with different excitation/emission spectra, images were acquired sequentially for each channel (488 nm, green and 555 nm, red) and the filters and photomultiplier tubes settings chosen to optimize images and avoid signal crosstalk. The prepared images were obtained from a maximum intensity Z-projection. In all images illustrating coronal or sagittal sections, the limits of the central canal (cc) or 4^th^ ventricle (4V) are represented with white dashed lines. Low resolution images of full sections (20x objective; generally 4×6 images/mosaic) were also acquired to allow identification of the section’s position in the rostro-caudal axis using specific anatomical landmarks and to orient each section in the dorsal-ventral axis.

### Morphology Analyses and Cell Counting

All analyses, cell counting and determination of markers co-expression were carried out on single images from stacks with cell to cell identification. The analyses were performed on non consecutive sections. Images were analyzed and prepared using ZEN 2009 light Edition (Zeiss software), ImageJ 1.45 (NIH), Adobe Illustrator and Photoshop. No correction was applied to the images and for a better visualization, especially for thin structures like fibers, only contrast and brightness were adjusted in the images used for the figures. When images of a same set of experiments are illustrated, the same adjustments were applied to allow comparison.

**Figure 2:**
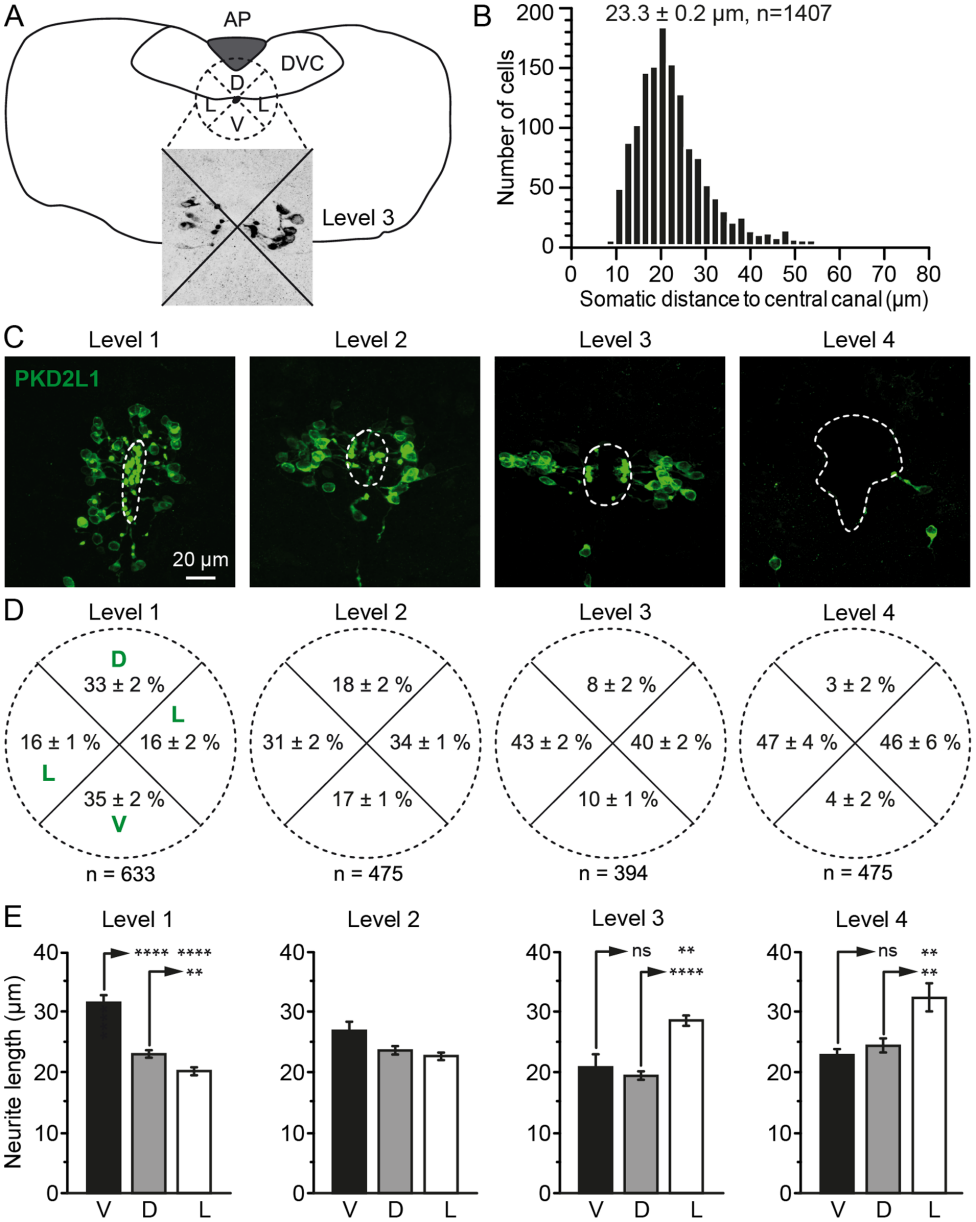
S-CSF-cNs are differentially distributed and organized along the central canal. A. Drawing of a brainstem coronal section at level 3 with the template of the 4 quarters (dashed cross and circle) used to analyze the distribution of PKD2L1^+^ cells around the cc (black dot). Inset shows PKD2L1^+^ cells distributed in the 4 quarters (black cross). **B.** Frequency histogram for the number of PKD2L1^+^ CSF-cNs according to the distance in µm between the soma and the border of the cc. Data are presented in 2 µm bins and the mean distance and number of cells are indicated on top of the graph. **C.** Representative coronal sections at level 1 to 4 illustrating the density and distribution of PKD2L1^+^ cells around the cc. **D.** Average percentage of PKD2L1^+^ cells distributed in the ventral, lateral and dorsal quarters around the cc (see panel A). **E.** Summary bar graphs of the average neurite length for S-CSF-cNs located in the ventral (V), dorsal (D) or lateral (L) regions and according to the medullospinal level (from left to right: level 1 to 4, respectively; ns: not significant; **, P<0.01 and ****P<0.0001). Data from both lateral regions were pooled together.

Cell density, distribution and morphology. At each medullospinal level of interest (see above and fig. 1D), the analysis was typically repeated in 3 animals and carried out for each animal and each of the 4 levels in 3 different sections (i.e. a total of 9 sections per level). For each section, PKD2L1^+^ cells were counted manually in every image within the corresponding image stack (14 to 24 images/stack and 15 to 26 µm stack depth) using the ‘cell counter’ ImageJ plug-in. Subsequently, the total number of cells counted was divided by the thickness of the analyzed stack and cell density expressed per 10 µm of tissue depth. To analyze the distribution of PKD2L1^+^ cells around the cc, each stack of images was oriented in the dorsal-ventral axis using the corresponding low resolution image. The images were then subdivided in 4 quarters centered on the cc as illustrated in Figure 2A, and for each image through the stack, cells were counted in each quarter using the ‘cell counter’ ImageJ plug-in. In all analyses, we refer to lateral orientation relative to the central canal, and did not distinguish between the animal’s left and right side. This procedure was carried out for each section and level and the average percentage of the cells distribution calculated for each quarter. The position of somas relative to the cc was measured as the distance between the soma of PKD2L1^+^ S-CSF-cNs and the border of the cc (straight line). To determine the neurite length of S-CSF-cNs depending on their position in a specific quarter and a specific level (see above for details), a segmented line (ImageJ) was used to follow over several optical slices the path of the neurite from the soma to the bud.

Cell counting in double labeling experiments. In experiments where we quantified the co-expression of selected cytoplasmic markers in PKD2L1^+^/EGFP^+^ S-CSF-cNs (GAD67, GABA, DCX and HuC/D), we manually counted the number of cells expressing PKD2L1/EGFP and the number of cells expressing the marker of interest. Subsequently, we calculated the percentage of PKD2L1^+^ cells co-expressing both markers. In experiments where the investigated marker was nuclear (NeuN except Nkx6.1), we first created a ROI at the size of a S-CSF-cN nucleus using ImageJ. This ROI was then used to measure in each image of a stack both the background average intensity (average of the pixel intensity for the ROI area; range 0–255) in a region without cell bodies or fibers (12.1±0.6 and 8.2±0.6 for NeuN experiments, in 9 sections in total) and the nuclear average intensity in all PKD2L1^+^ cells (27.7±1.5, 673 cells; P<0.001against the background signal for NeuN experiments). Out of the values obtained for each image in a determined stack, we then calculated the mean background intensity and only PKD2L1^+^ cells with an average nuclear labeling intensity greater than 30% of the background value were considered positive for NeuN.

### Statistics

All data are expressed as mean ± S.E.M and the statistical significance was tested using a Kruskal-Wallis test (non-parametric) combined with a Dunn’s multiple comparison tests. Data were considered to be significantly different given a P value below 0.05. Analyses were carried out using Excel 2007 (Microsoft) and Prism (Graphpad).

## Results

### Characterization of S-CSF-cNs Along the Central Canal of Adult Mice

**Figure 3:**
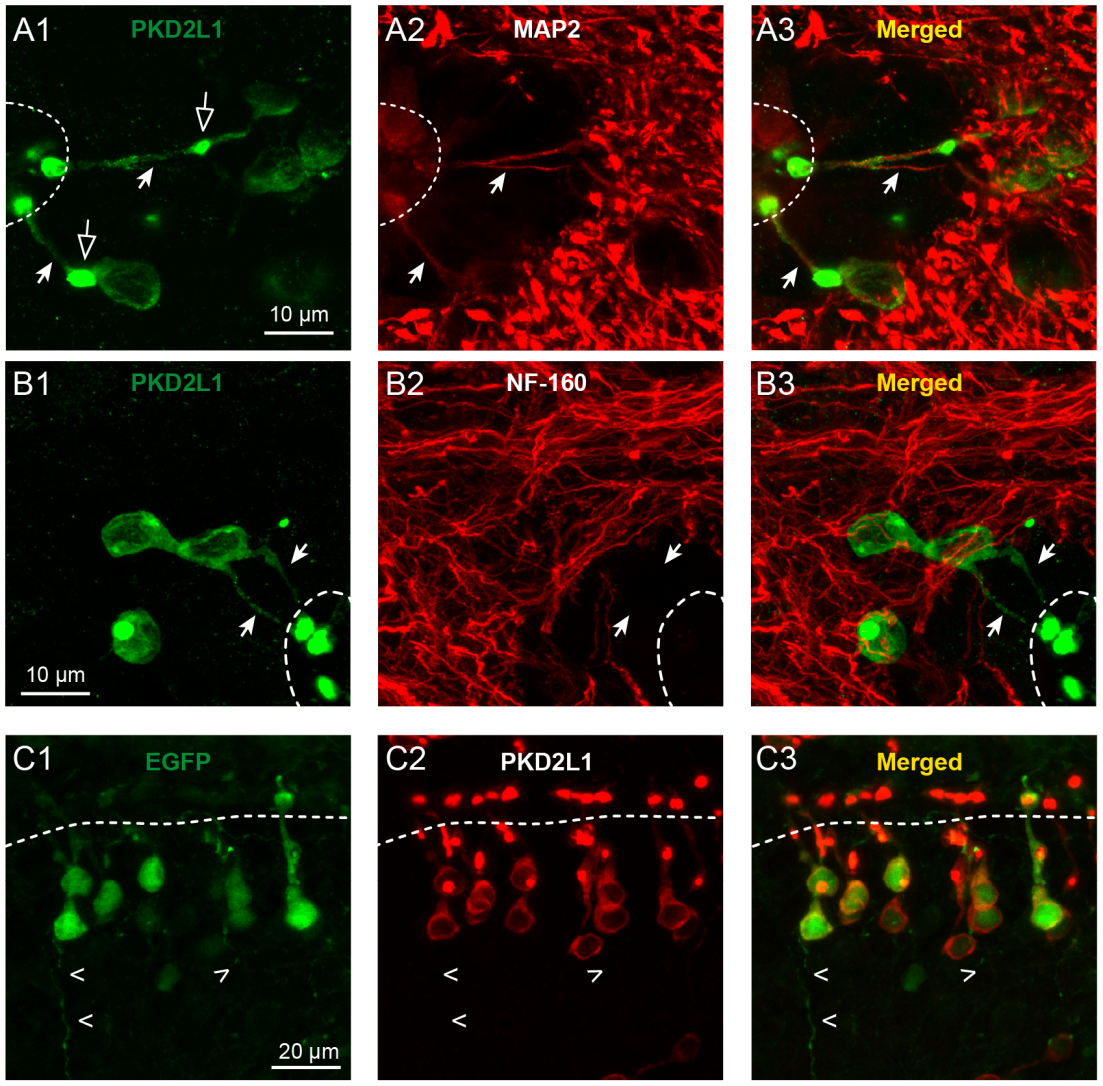
S-CSF-cNs are polarized and express PKD2L1 on dendrite and soma but not on the axon. A1 and B1. Immunolabeling of S-CSF-cNs in medial brainstem coronal sections with antibodies against PKD2L1 (green) and either MAP2 (**A2**, red) or NF-160 (**B2**, red) to illustrate the polarity of PKD2L1^+^ neurons. These cells project a thick neurite (arrows) to the cc with a cytoplasmic enlargement (open arrows) and terminated by a PKD2L1^+^ terminal protrusion or bud. **A3.** The merged image shows the superimposition of PKD2L1 (green) and MAP2 (red) immunoreactivity and indicates that S-CSF-cNs project a dendrite (MAP2^+^) to the cc. Note that no MAP2 positive and PKD2L1 negative projections or cells are observed in the ependymal layer. **B3.** The merged image of the PKD2L1 (green) and NF-160 (red) immunoreactivity indicates the presence of numerous NF-160^+^ axon-like fibers in the section, around the cc and over S-CSF-cNs and show that the thick projection to the cc is not NF-160 positive (arrows). However it is difficult to visualize axons leaving the somata of PKD2L1^+^ S-CSF-cNs. **C.** Immunohistochemistry against GFP (**C1**) and PKD2L1 (**C2**) in a sagittal brainstem section obtained from a PKD2L1:EGFP^+^ mouse to visualize axonal projections and the PKD2L1 distribution in CSF-cNs. **C3.** The superimposed image of anti-GFP (green) and anti-PKD2L1 (red) immunoreactivity shows that CSF-cNs do present a thin axon-like structure leaving the soma towards the parenchyma (open arrowheads in C) but PKD2L1 is exclusively expressed on the somatodendritic compartment.

Morphology and distribution of PKD2L1^+^ S-CSF-cNs along the central canal. Using an immunohistochemical approach with primary antibodies directed against PKD2L1, we first specified the morphology of PKD2L1^+^ CSF-cNs and compared it along the length of the medullospinal cc on coronal sections from cervical spinal cord and brainstem. Figure 1A illustrates representative coronal sections at the level of the cervical spinal cord (level 1, fig. 1A, Left and see Methods Section) and caudal brainstem (level 2, fig. 1A, Right) where PKD2L1^+^ cells can be observed around the cc. Typically, the large majority of PKD2L1^+^cells (93.2±0.8% with 1549 out of 1703 PKD2L1^+^ cells analyzed) presented the classical morphology described in mammals for CSF-cNs around the cc (fig. A1 arrowheads and B) [5], [8], [11], [12], [31]. Thus, PKD2L1^+^ cells had a small soma with an average diameter of 8.0±0.1 µm (336 cells) and projected a thick neurite (see below) to the cc that ended with a protrusion or bud (average diameter of 3.1±0.1 µm; 315 cells). We further noticed that PKD2L1^+^ CSF-cNs regularly exhibited an enlargement along the thick neurite (figs. 1B and 3A1, open arrows) that was most visible on sagittal sections (see figs. 3C1 and 4). In some instances, we also observed PKD2L1^+^ cells presenting a bipolar morphology with thin processes that did not clearly appear to be directed at and/or in contact with the cc (fig. 1A, arrows and C). Since in the present study we aimed at determining and analyzing the distribution and phenotype of CSF-cNs expressing PKD2L1, we excluded this latter population of PKD2L1^+^ cells from our analysis to focus on cells exhibiting the classical morphology of CSF-cNs.

**Figure 4:**
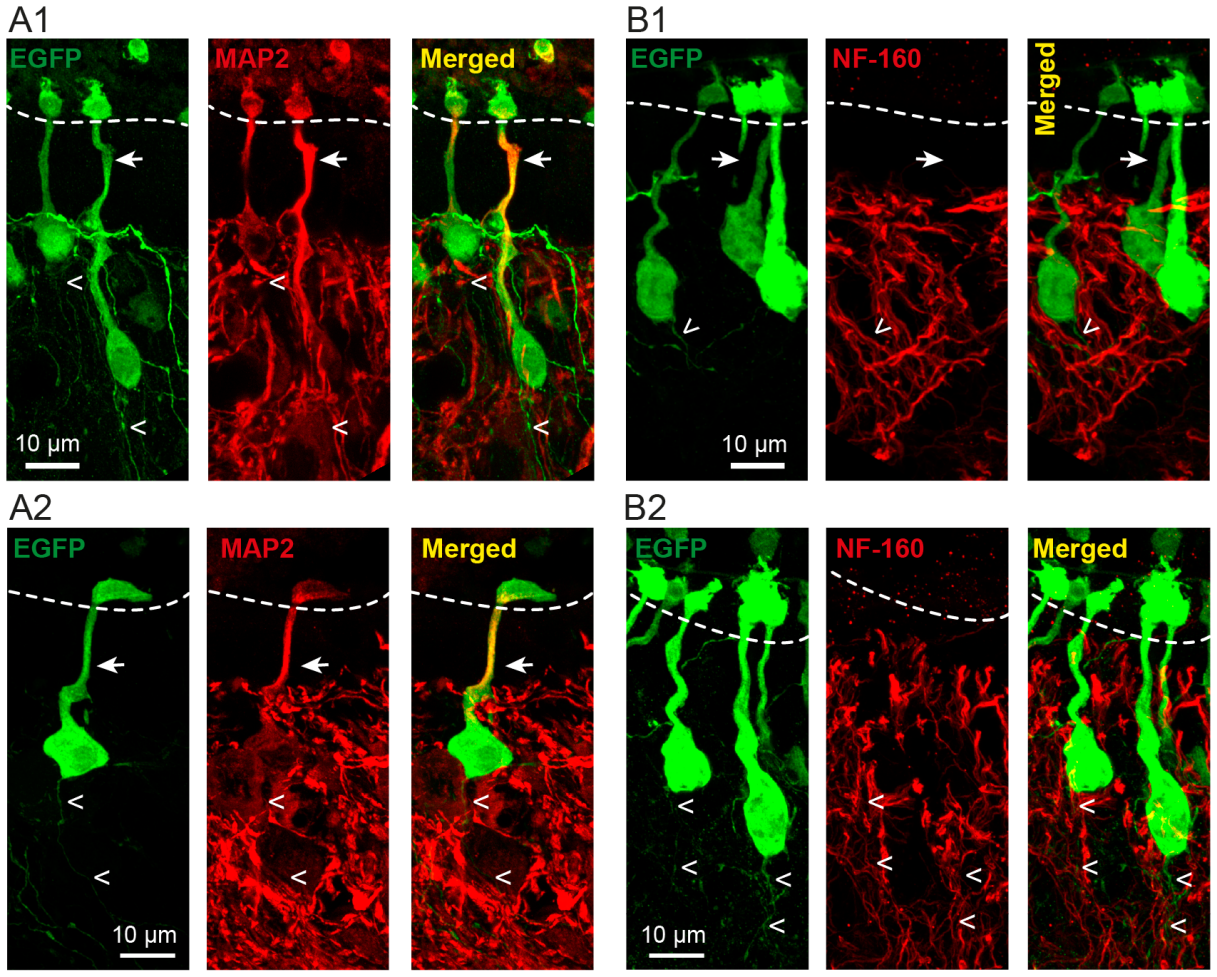
S-CSF-cNs project a dendrite towards the central canal and an axon-like fiber through the parenchyma. A1 and 2. Immunolabeling of S-CSF-cNs with antibodies against GFP (green) and MAP2 (red) in two different brainstem sagittal sections obtained from PKD2L1:EGFP^+^ mice. Immunostaining against EGFP (Left in A1 and 2 and see also B1 and 2) reveals the whole S-CSF-cNs cell morphology with, on one side, a thick neurite projecting to the cc and ending with a bud and, on the other side, the presence of thin fiber structures leaving the soma through the parenchyma. The merged images (Right) show that the thick neurite sent to the cc is labeled by antibodies against MAP2 while the thin fibers in the parenchyma are not. Note the presence of cilia-like structure on top of the bud in A1, B1 and B2. **B1 and 2.** Immunolabeling of S-CSF-cNs in two different brainstem sagittal sections obtained from PKD2L1:EGFP^+^ mice with antibodies against GFP (green) and NF-160 (red). Immunostaining against NF-160 (Middle) show that the thick MAP2^+^ neurites are not labeled by NF-160 antibodies and thus confirmed the dendritic nature of this projection. NF-160 immunolabeling is observed in the parenchyma with the presence of numerous fibers. However, the merged images (Right) shows that the thin fibers emerging from the soma of S-CSF-cNs are not NF-160^+^. Arrows: point to thick dendrite; open arrowheads: point to thin axon-like fibers.

We next examined the localization and distribution of PKD2L1^+^ CSF-cNs around the cc in the cervical spinal cord (level 1) and through the brainstem (levels 2 to 4; fig. 1D and see Methods Section). As shown on the representative sagittal section illustrated in Figure 1F, PKD2L1^+^ CSF-cNs were present along the length of the cc but their density progressively decreased from level 1 (most caudal) to 4 (most rostral). Indeed, when counting PKD2L1^+^ CSF-cNs per 10 µm of tissue depth on images obtained from coronal sections, we found: 10±1 cells at level 1, 8±1 and 7±1 at levels 2 and 3, respectively and only 3±1 at level 4 (fig. 1E; P<0.05). Our data also indicated that PKD2L1^+^ CSF-cNs presented a homogeneous morphology with a subependymal localization at all analyzed levels and that no PKD2L1^+^ cells were observed in the ependymal layer (*i.e.* no intraependymal CSF-cNs; see below and figs. 3A and 4). Further, the average distance between their soma and the cc was 23.3±0.2 µm (fig. 2B, n = 1407). In the rest of the study, we therefore refer to PK2DL1^+^ cells as subependymal CSF-cNs or S-CSF-cNs.

When analyzing the localization of S-CSF-cNs around the cc depending on the level (1 to 4) along the rostro-caudal axis, we noticed that PKD2L1^+^ S-CSF-cNs were differentially organized in the dorsal-ventral axis (*i.e.* lateral; see Methods Section and fig. 2A, C and D; P<0.05). Thus at level 1, PKD2L1^+^ S-CSF-cNs were principally present in a ventral (on average 35±2%) and dorsal (on average 33±2%) position with less cells present laterally (on average 16±1%; fig. 2C and D). Figure 2D (from Left to Right) indicates that this organization progressively changed with a shift to a lateral position at the more rostral levels (on average from 46±6% to 47±4% laterally and only 4±2% and 3±2% ventrally and dorsally, respectively at level 4; fig. 2C and D; P<0.05). Our data also indicated that S-CSF-cNs with a ventral localization at level 1 and 2 and with a lateral one at level 3 and 4 had the longest neurites (fig. 2C and D). Indeed at level 1, the neurites of ventral S-CSF-cNs was 31.7±1.3 µm long (n = 81) while it was only around 20 µm in the other regions around the cc (dorsal: 23.0±0.4, n = 107; lateral: 20.3±0.6, n = 114; P<0.0001; fig. 2E, Left). Along the rostro-caudal axis (from Left to Right; fig. 2E) the neurite length of ventrally located PKD2L1^+^ S-CSF-cNs progressively decreased down to 23±0.4 µm (n = 15) and in parallel the neurite length of lateral S-CSF-cNs increased to 32.5±2.3 µm (n = 22) at level 4 (dorsal: 24.5±1.0, n = 14; P<0.01; fig. 2E, Right).

**Figure 5:**
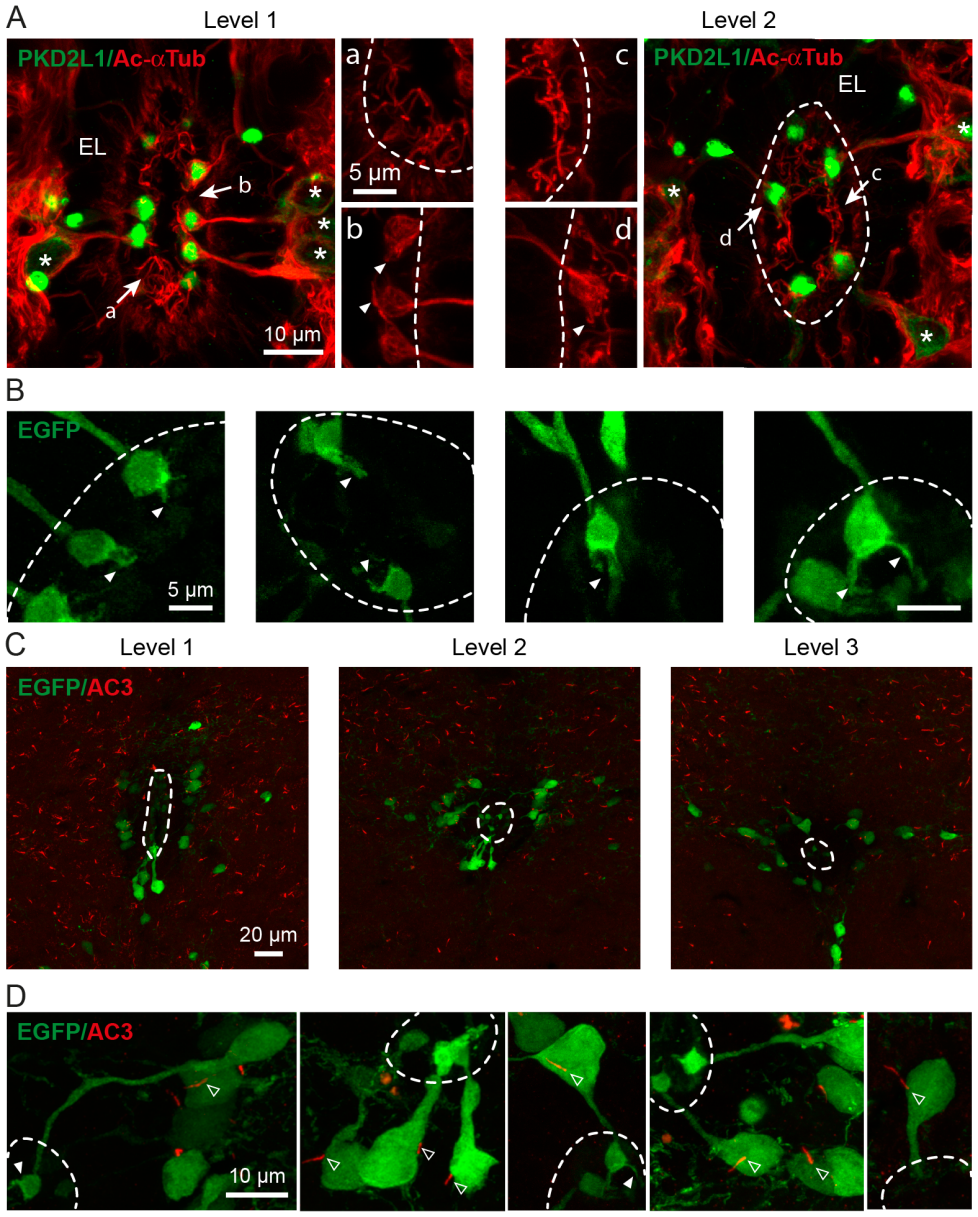
S-CSF-cNs present ciliary structures on the bud and primary cilia arising from the soma. A. Sections obtained from level 1 and 2, are immunolabeled with antibodies against PKD2L1 (Green) to reveal S-CSF-cNs and with acetylated α tubuline (Ac-αTub, Red), a selective marker of the cytoskeleton and ciliary structures. The merged images indicate that the soma (stars), the dendrite and the bud of PKD2L1^+^ S-CSF-cNs are positively labeled by Ac-αTub and also reveal the presence of numerous cilia in the lumen of the cc most certainly arising from ependymocytes. **Aa to d.** At higher magnification, Ac-αTub^+^ cilia can be observed in the lumen of the cc (a, level 1 and c, level 2) but small cilia-like appendices are also visualized on the bud of S-CSF-cNs (b, level 1 and d, level 2; arrowheads). **B.** High magnification images of buds from S-CSF-cNs in sections obtained from PKD2L1:EGFP^+^ adult mice and immunolabeled against GFP indicate the presence of a cilia-like structure or tuft of cilia at the end of the bud (arrowheads; see also figs. 3 and 4). **C.** Coronal sections from levels 1 to 3 obtained from a PKD2L1:EGFP^+^ adult mice showing the results of immunostaining with antibodies against GFP (green) and AC3 (red). Note the presence of AC3^+^ labeling on GFP^+^ S-CSF-cNs but also throughout the parenchyma (thin red rods). **D.** High magnification images showing the presence of AC3^+^ cilium (open arrowheads) on the soma of GFP^+^ S-CSF-cNs. Note that no AC3^+^ immunoreactivity is observed on the bud although some GFP^+^ ciliary structures can be observed (arrowheads).

PKD2L1 is expressed on the somatodendritic compartment of S-CSF-cNs. In order to assess the cellular localization of PKD2L1 proteins, we first determined the cell polarity of PKD2L1^+^ S-CSF-cNs and identified the somatodendritic versus axonal compartments using subcompartment-specific antibodies (figs. 3 and 4). Using anti-MAP2 antibodies, we showed that the thick neurite projecting towards the cc was MAP2^+^ and thus of dendritic nature (fig. 3A2 and A3). This dendrite strongly co-expressed PKD2L1 with the highest level of immunoreactivity on the terminal bud (fig. 3A1 and A3). Interestingly, these data also indicated a complete absence of MAP2^+^ cells in the ependymal layer and suggested that all MAP2^+^ cells projecting to the cc were PKD2L1^+^ S-CSF-cNs (see figs. 3–5). The dendritic nature of this thick neurite was further confirmed by the absence of immunolabeling against the subtype of neurofilaments with a molecular weight of 160,000 (NF-160), a selective marker for axons (fig. 3B, arrows). These results also showed the presence of numerous labeled axonal fibers in brainstem coronal sections around the cc and over S-CSF-cNs, but we could not identify axon-like NF-160^+^ fibers arising from the soma of S-CSF-cNs that were also PKD2L1^+^ (fig. 3B1 and B3). We therefore looked for axons in sagittal sections obtained from PKD2L1:EGFP^+^ mice where EGFP would fill the cytoplasm of S-CSF-cNs thus enabling the visualization of their axon. After labeling cytoplasmic EGFP with a selective antibody against GFP, we did indeed observe the typical morphology of S-CSF-cNs with thick dendrites projecting to the cc (fig. 3C and arrows in fig. 4) and a thin axon-like process running through the parenchyma (open arrowheads in figs. 3C and 4). The comparative analysis of PKD2L1^+^ immunoreactivity on EGFP^+^ S-CSF-cNs showed that PKD2L1 was expressed on the soma and dendrite of S-CSF-cNs (fig. 3C2) but not on their axon-like process (fig. 3C2 and C3, open arrowheads).

To examine further the polarity of S-CSF-cNs, we repeated immunolabeling experiments against either MAP2 (fig. 4A) or NF-160 (fig. 4B) on similar brainstem sagittal sections obtained from PKD2L1:EGFP^+^ mice. Our results confirmed that the thick neurites were positively labeled against MAP2 (fig. 4A, arrows) but not against NF-160 (fig. 4B, arrows) and that the thin processes running ventrally through the parenchyma were MAP2 negative (fig. 4A, open arrowheads). Although antibodies against MAP2 failed to label the thin GFP^+^ processes in the parenchyma (fig. 4A, open arrowheads), neither were these structures labeled by antibodies against NF-160 (fig. 4B, open arrowheads). While we thus did not immunohistochemically confirm the axonal identity of these fibers, based on their morphology we chose to refer to these structures as axon-like processes (and see below).

Medullar S-CSF-cNs possess a primary cilia on their soma. In spinal cord, the presence of cilia on the bud of CSF-cNs has been reported in several vertebrate species [4], [8], [11], [32] but to our knowledge the nature of this cilium, primary versus motile cilium, has not been demonstrated. We first carried out double labeling experiments in cervical spinal cord and brainstem coronal sections using primary antibodies against PKD2L1 and acetylated α-tubuline (Ac-αTub), a marker for microtubule-based cilia (fig. 5). Figure 5A shows that Ac-αTub strongly labeled not only PKD2L1^+^ S-CSF-cNs (stars) revealing their classical morphology but also numerous ciliary structures around the cc (fig. 5A). Interestingly, at higher magnification, we could visualize thin cilia in the cc arising from the edge of the cavity and most certainly corresponding to the motile cilia of ependymocytes (fig. 5Aa and c). A closer examination of PKD2L1^+^ S-CSF-cNs suggested the presence of Ac-αTub^+^ ciliary-like structures on their bud (fig. 5Ab and d, arrowheads) that appeared to be PKD2L1 negative. This result was further supported by the data obtained following immunolabeling against GFP, as illustrated in Figure 5B (arrowheads), where ciliary-like structures could be observed on top of the bud (see also figs. 3C1 and 4). Since adenylate cyclase 3 (AC3) is a prominent marker of primary cilia in the brain [33], we carried out AC3/GFP double-labeling on cervical spinal cord and brainstem coronal sections obtained from PKD2L1:EGFP^+^ mice to determine whether the structures observed on the bud were primary cilia (fig. 5C and D). We did observe clear AC3 labeling of small rods all over the parenchyma and around the cc in coronal sections at all levels (fig. 5C). Figure 5D illustrates representative GFP^+^ S-CSF-cNs exhibiting AC3 labeled cilia on their soma (White open arrowheads) but not on the bud (White arrowheads). We observed these primary cilia on the soma of all PKD2L1:EGFP^+^ S-CSF-cNs with an average length of 3.4±0.2 µm (n = 153) and a diameter in the sub-micrometer scale.

Taken together, the results presented above indicated that PKD2L1^+^ CSF-cNs are present along the length of the cc with an exclusive subependymal position (S-CSF-cNs). They exhibit a conserved morphology with a dendrite projecting to the cc that ends with a protrusion that appears to present a ciliary structure; they also exhibit a primary cilium on their soma. Furthermore, these cells strongly express PKD2L1 only on their somatodendritic compartment and this protein appeared to be a specific maker for this cell population. Finally, the analysis of their distribution from spinal cord to brainstem indicates that their density decreases and that both their position around the cc and the length of their dendrite changes along the rostro-caudal axis.

### Phenotype of Brainstem PKD2L1^+^ S-CSF-cNs

**Figure 6:**
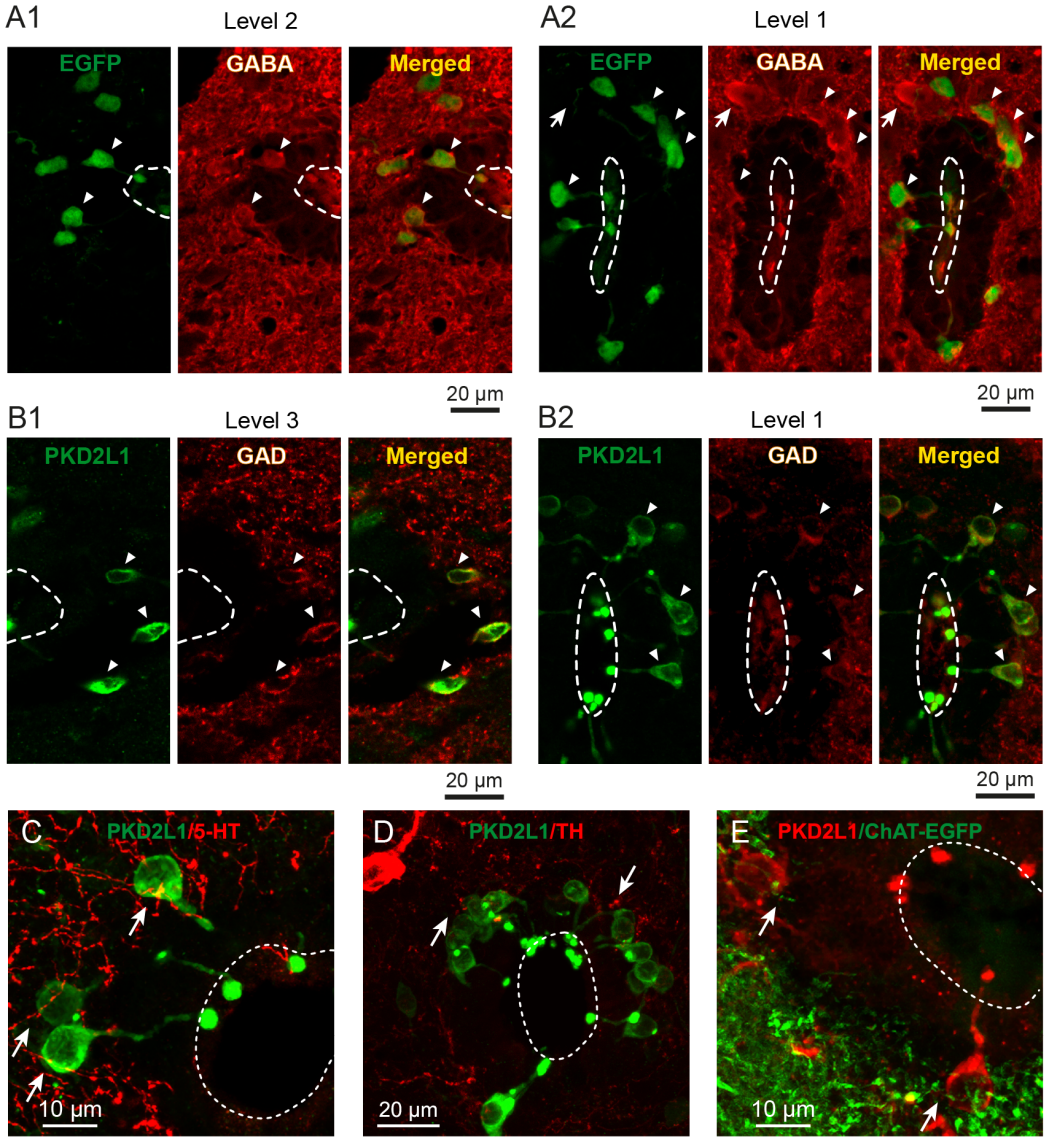
PKD2L1^+^ CSF-cN are GABAergic and in close contact with GABAergic, cholinergic and aminergic fibers. A1. Immunolabeling of S-CSF-cNs with GFP (Left) and GABA (Middle) antibodies in a brainstem coronal section (level 2). The merged image (Right) shows superimposition of the immunoreactivity against GFP (green) and GABA (red) and indicates that most medullar GFP^+^ S-CSF-cNs are also positive for GABA (arrowheads). **A2.** Similar results are visualized in a section at level 1 where spinal GFP^+^ S-CSF-cNs (Left, green) are also positive for GABA (Middle, red and Left, merged). **B1.** Immunolabeling of S-CSF-cNs with PKDL1 (Left) and GAD67 (Middle) antibodies in a brainstem coronal section (level 3). The merged image (Right) indicates that most medullar PKD2L1^+^ S-CSF-cNs are also GAD67^+^ (arrowheads). **B2.** Again, similar results are visualized in a section at level 1 where spinal S-CSF-cNs coexpress PKD2L1 with GAD67. Note that the GAD67 immunolabeling is present in the cytoplasm of S-CSF-cNs (arrowheads in B1 and 2) as well as in the parenchyma (red punctae). The immunolabeling against 5-HT (**C**, red) and TH (**D**, red) shows that PKD2L1^+^ S-CSF-cNs (green) in the brainstem are neither serotoninergic nor catecholaminergic. In **E**, labeling with anti-PKD2L1 antibodies in a brainstem coronal section prepared from choline-acetyltransferase (ChAT):EGFP adult mice indicates that PKD2L1^+^ S-CSF-cNs (red) are not cholinergic (green). Note that PKD2L1^+^ S-CSF-cNs appear to be in close contact with serotoninergic (**C**), catecholaminergic (**D**) and cholinergic (**E**) fibers (arrows).

Earlier studies have indicated that spinal CSF-cNs were GABAergic in rodents [6], [11]. To demonstrate the phenotype of medullar S-CSF-cNs in mice, we carried out GABA/GFP double labeling on sections obtained from PKD2L1:EGFP^+^ mice. The selectivity of the GABA/GAD67 immunolabeling was confirmed by the presence of GABA^+^/GAD67^+^ cells in brain regions known to contain GABAergic neurons: the spinal cord dorsal horn, the *nucleus tractus solitari* and the cerebellum (Data not shown). As visualized in Figure 6A1, a large proportion of medullar GFP^+^ S-CSF-cNs were also GABA^+^ (86±3% of GABA and PKD2L1^+^ positive cells; 124 GABA^+^ cells out of 140 GFP^+^ S-CSF-cNs; fig. 6A1, arrowheads). Similar results were observed at level 1 where 90±2% of GFP^+^ S-CSF-cNs were also GABA^+^ (118 GABA^+^ cells out of 131 GFP^+^ S-CSF-cNs; fig. 6A2, arrowheads). Moreover, when considering all analyzed levels, we found that 88±2% of GFP^+^ S-CSF-cNs were also GABA^+^ (242 GABA^+^ cells out of 271 GFP^+^ S-CSF-cNs).

We further confirmed the GABAergic phenotype for S-CSF-cNs, by carrying out a double immunolabeling analysis against GAD67 and PKD2L1. And indeed, as illustrated in Figure 6B, our results indicated that 78±3% of PKD2L1^+^ medullar S-CSF-cNs coexpressed GAD67 (286 GAD^+^ cells out of 356 PKD2L1^+^ S-CSF-cNs; fig. 6B1 for level 3). The same analysis showed that at level 1 (fig. 6B2) 86±2% of spinal PKD2L1^+^ S-CSF-cNs were also GAD67^+^ (210 GAD^+^ cells out of 250 PKD2L1^+^ S-CSF-cNs) and that overall 79±2% of S-CSF-cNs were PKD2L1^+^/GAD67^+^ (level 1–4∶577 GAD^+^ cells out of 733 PKD2L1^+^ S-CSF-cNs). In these cells we primarily observed GAD67^+^ staining delineating the soma and the bud of PKD2L1^+^ S-CSF-cNs (arrowheads in fig. 6B). Additionally, numerous GAD67^+^ dots were present all over the analyzed sections with some of them sitting close to or on the soma of PKD2L1^+^ S-CSF-cNs (fig. 6B). Although S-CSF-cNs thus appeared largely GABAergic, around 15% were GABA/GAD67 negative. We therefore tried to identify the phenotype of the remaining S-CSF-cNs. However, none of the PKD2L1^+^ S-CSF-cNs were serotoninergic (5-HT; fig. 6C) or catecholaminergic (TH; fig. 6D). The remaining PKD2L1^+^ S-CSF-cNs were not cholinergic either as no coexpression of PKD2L1 and EGFP was observed in brainstem sections prepared from ChAT:EGFP mice (fig. 6E).

Our results indicated that medullar S-CSF-cNs, like their spinal counterparts, are mostly GABAergic. Furthermore, they appeared to be surrounded by GAD67 positive terminals and to be in close contact or apposition with cholinergic and monoaminergic fibers (arrows in fig. 6C to E).

### Medullar PKD2L1^+^ CSF-cNs Express Immature Neuronal Markers and Nkx6.1

In young animals, CSF-cNs around the cc were shown to express markers of immature neurons and to be NeuN negative [8]. In adult animals, most available information regarding the maturity of the peri-canal region of the spinal cord concerns cells located within the ependymal layer [22], [23], [26], [31] and little is known about the medullar CSF-cNs population. We therefore carried out a set of experiments to quantitatively assess the degree of maturity of PKD2L1^+^ S-CSF-cNs in the brainstem and compared it to the situation observed in the cervical spinal cord.

**Figure 7:**
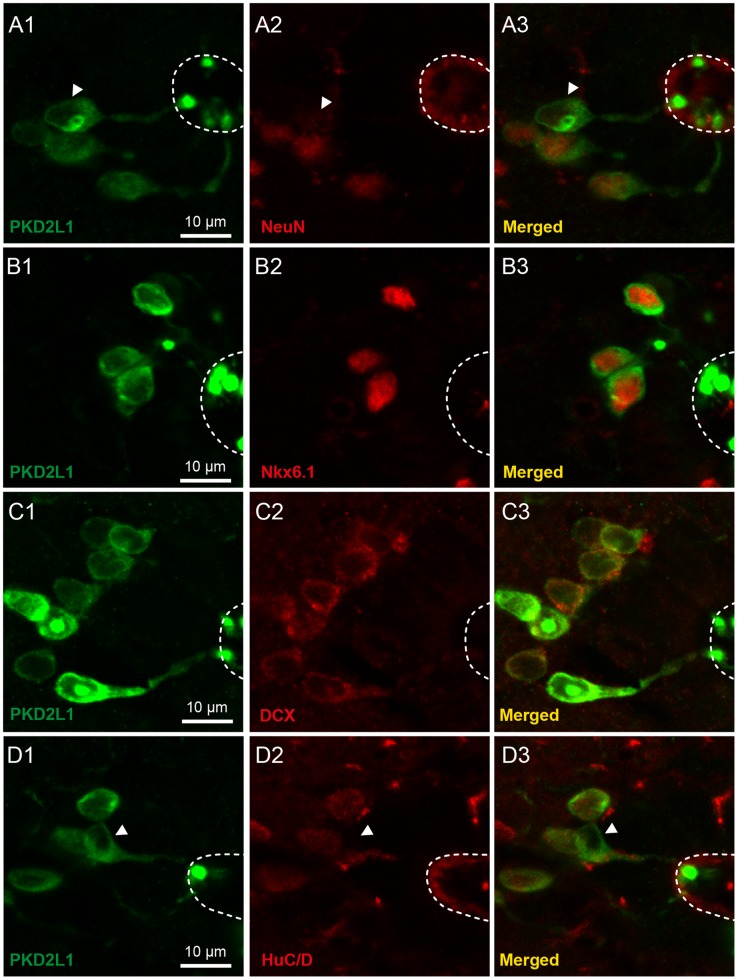
In adult mice S-CSF-cNs express NeuN along with classical markers of neuronal immaturity. A1 to D1. Immunolabeling of S-CSF-cNs in medial brainstem section (level 2) prepared from the same animal with antibodies against PKD2L1 (green) and either NeuN (**A2**, red), Nkx6.1 (**B2**, red), DCX (**C2**, red) or HuC/D (**D2**, red). **A3.** The merged image shows that out of 4 PKD2L1^+^ S-CSF-cNs only one is NeuN negative (arrowheads in A1 to 3). **B3.** The merged image shows that all PKD2L1^+^ S-CSF-cNs are also positive for Nkx6.1. Note the strong immunolabeling of nuclei in S-CSF-cNs. In **C3**, the merged image of PKD2L1 and DCX immunoreactivity shows that all PKD2L1^+^ S-CSF-cNs are DCX^+^. The superimposed image in **D3** shows that only one PKD2L1^+^ S-CSF-cN is negative for HuC/D (arrowheads in D1 to 3).

**Figure 8:**
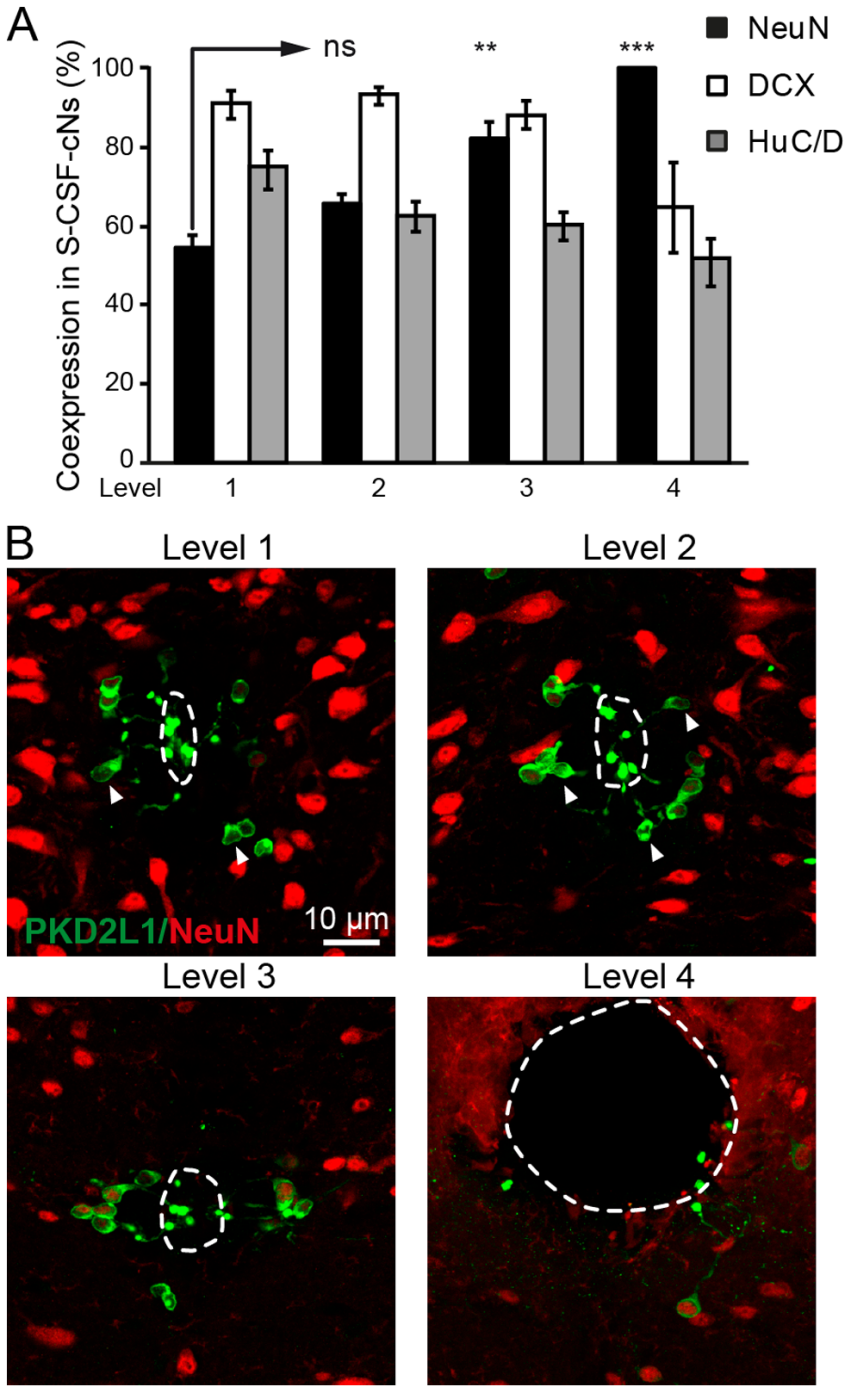
Expression pattern for maturity and immaturity markers in S-CSF-cNs along the central canal. A. Summary bar graph of the average co-expression percentage of NeuN (black bars), DCX (white bars) and HuC/D (gray bars) in PKD2L1^+^ S-CSF-cNs along the rostro-caudal axis of the cc (level 1 to 4; see Methods Section and Text for details). The data indicate that NeuN expression increased in S-CSF-cNs along the rostro-caudal axis while DCX expression pattern remained similar at all levels. HuC/D expression showed a tendency for a decrease along the rostro-caudal axis. Total number of analyzed PKD2L1^+^ S-CSF-cNs for each markers: NeuN, 296 cells at level 1; 208 cells at level 2; 153 cells at level 3 and 16 cells at level 4. DCX: 213 cells at level 1; 156 cells at level 2; 138 cells at level 3 and 17 cells at level 4. HuC/D: 173 cells at level 1; 144 cells at level 2; 118 cells at level 3 and 26 cells at level 4. ns: not significant; **, P<0.01 and ***P<0.001. **B.** Merged images showing the superimposition of PKD2L1 (green) and NeuN (red) immunoreactivity in sections from level 1 to 4 and illustrating the increase in the percentage of PKD2L1^+^ S-CSF-cNs expressing NeuN. PKD2L1^+^ S-CSF-cNs that are negative for NeuN are indicated by arrowheads.

We first looked for the expression of NeuN, a classical marker for mature neurons, in PKD2L1^+^ S-CSF-cNs of 3-month-old animals. In the caudal brainstem (level 2), we found that on average 66±3% of PKD2L1^+^ S-CSF-cNs expressed NeuN (262 NeuN^+^ cells out of 361 PKD2L1^+^ S-CSF-cNs; figs. 7A and 8) although with a ∼4 times lower apparent intensity of labeling than that of the PKD2L1 negative neighboring neurons (cell body average NeuN^+^ labeling intensity: 100±5, 100 cells in the parenchyma and 28±2 in 262 PKD2L1^+^ S-CSF-cNs; P<0.0001). Further, our results indicated that the proportion of PKD2L1^+^ S-CSF-cNs expressing NeuN progressively increased from 54±4% at level 1 to 100% in the most rostral region of the cc (level 4; fig. 8, P<0.001). Interestingly, the apparent intensity of the immunolabeling in S-CSF-cNs also appeared to progressively increase following the rostro-caudal axis (fig. 8B).

Next, using sections obtained from the same animals, we assessed the expression level and pattern of markers indicative of neuronal immaturity. In the spinal cord of young rodents, cells with the typical morphology of CSF-cNs were shown to express Nkx6.1, a homeodomain protein present in differentiating neurons of the ventral spinal cord, and several other classical markers of neuronal immaturity [8], [11], [22], [31]. As illustrated in Figure 7B, medullar PKD2L1^+^ S-CSF-cNs of 3-month-old mice strongly expressed Nkx6.1 (98±1%; 52 Nkx6.1^+^ out of 53 PKD2L1^+^ S-CSF-cNs at level 2). The entire population of PKD2L1^+^ S-CSF-cNs from the cervical spinal cord up to the rostral part of the brainstem also strongly expressed Nkx6.1 (99±1% of cells coexpressed Nkx6.1 and PKD2L1 when considering all 4 levels; 111 Nkx6.1^+^ cells out of 112 PKD2L1^+^ cells). Further, using a selective antibody against DCX, we also demonstrated that 90±1% of medullar PKD2L1^+^ S-CSF-cNs (level 2) coexpressed DCX (277 DCX^+^ cells out of 294 PKD2L1^+^ S-CSF-cNs; figs. 7C and 8A) and that a similar level of DCX expression was found at all analyzed regions of the cc (94±2%; 468 DCX cells out of 524 PKD2L1^+^ S-CSF-cNs; fig. 8A). We also reported that 63±4% of medullar PKD2L1^+^ S-CSF-cNs (level 2) expressed HuC/D (159 HuC/D^+^ cells out of 262 PKD2L1^+^ S-CSF-cNs; figs. 7D and 8A). In young rats, immunoreactivity against PSA-NCAM was reported in the region surrounding the central canal [8], [31], [34]. We therefore tested, using selective antibodies, for the presence of PSA-NCAM expression in PKD2L1^+^ S-CSF-cNs of adult mice. Although, we did observe positive labeling for PSA-NCAM in the subventricular zone or the dentate gyrus of the hippocampus, in the same experimental conditions, we were unable to detect any labeling against PSA-NCAM (Data not shown). Finally, although the number of PKD2L1^+^ S-CSF-cNs expressing DCX appeared similar at all 4 levels, there was a tendency for a decrease in the number of S-CSF-cNs that expressed HuC/D along the rostro-caudal axis (fig. 8A).

The analysis presented above demonstrates that PKD2L1^+^ S-CSF-cNs express NeuN and HuC/D as well as DCX and Nkx6.1, two markers indicative of neuronal immaturity. Furthermore, following the rostro-caudal axis of the cc, it appears that the number of S-CSF-cNs expressing DCX is constant while the number of PKD2L1^+^/NeuN^+^ and that of PKD2L1^+^/HuC/D^+^ S-CSF-cNs appeared to increase and decrease, respectively.

## Discussion

### Morphology and Distribution of S-CSF-cNs Around the Central Canal

The aim of the present study was first to determine the morphology, distribution and localization of PKD2L1 expressing CSF-cNs along and around the cc of the cervical spinal cord (level 1) and the brainstem (level 2–4) in adult animals. Furthermore, we carried out a quantitative and comparative analysis of protein expression levels within these cells.

Using an immunohistochemical approach with antibodies against PKD2L1, we observed two groups of PKD2L1^+^ cells around the cc: one predominant population with the previously reported morphology for CSF-cNs [6]–[11] and a second, less numerous populations, comprised of cells presenting multiple thin processes with no clear projection towards the cc. We excluded this latter cell population to focus our analysis on cells presenting a clear morphology of medullospinal CSF-cNs [6], [7], [9]. We found MAP2 immunoreactivity along the entire length of the cc, but we observed no MAP2^+^ cells in the medullospinal ependymal layer of mice tissue, indicating the absence of intraependymal CSF-cNs. Secondly, we observed that all MAP2^+^ CSF-cNs coexpressed PKD2L1. We therefore concluded that, in mice, PKD2L1 represents a selective marker for medullospinal CSF-cNs and that these neurons have an exclusively subependymal localization.

We showed that along the length of the medullospinal cc, these cells represent a homogenous neuronal population exhibiting a simple morphology with a small round soma (∼10 µm) extending a single thick dendrite (MAP2^+^ and NF-160 negative) through the ependymal layer. This dendrite ends in the CSF cavity with a bud of conserved diameter (∼3 µm) on top of which, we observed, in some instances, a cilia-like structure (see below). Our data are in agreement with previous reports obtained in the mouse spinal cord [6], [9], [10]. However, they contrast with the results obtained from rats [8], [11] or lower vertebrates [20], [35], [36] where CSF-cNs were shown to be mainly intraependymal and to exhibit a pear-shaped soma extending throughout the whole width of the ependymocytes layer. We hypothesize that this result might be explained by differences between species or in the stage of development as suggested by a recent study carried out in rats [29]. We also regularly observed an enlargement along the thick dendrite that exhibited a strong immunoreactivity against PKD2L1. In the hypothalamus and the pineal gland, where CSF-cNs can also be found (not in the spinal cord or brainstem) a similar structure had been reported and was suggested to correspond to a lipid droplet [37], [38]. We do not have any evidence to support this hypothesis and further investigations would be necessary to determine the nature and role of this enlargement.

Furthermore, medullospinal CSF-cNs appear to be polarized, as they also send a thin axon-like process through the parenchyma that was best seen in sagittal sections using labeling against GFP. Note that PKD2L1, being an ion channel, is localized to specific parts of the membrane, while EGFP is classically cytosolic and therefore free to diffuse throughout the whole cell. These differences would explain why global cell morphology is best resolved following labeling against EGFP. Our data suggest that the thin processes are not dendrites, as they were MAP2 negative, but we were not able to confirm that they were axons, because they were also negative for NF-160, a selective marker for intermediate filaments of mature axons [39], [40] (see also below). In rat spinal cord, Stoeckel and colleagues [11] suggested that axons of CSF-cNs would form ventral fiber tracts that would run longitudinally at the level of the median fissure. Correspondingly, the thin axon-like projection that we observed appear to run ventrally but they were difficult to visualize in all S-CSF-cNs and we did not determine their destination. We know very little about the targets of these axon-like processes [11], but identifying the regions that are innervated by S-CSF-cNs and the physiological role of these projections is an important future step.

We demonstrated that PKD2L1^+^ S-CSF-cNs are present at a high density (5–10 cells/10 µm of tissue) around the cc. We found the largest density at the cervical level of the spinal cord with a progressive decrease as we moved rostrally; fewer PKD2L1^+^ S-CSF-cNs were observed around the 4V. We also found that the localization of PKD2L1^+^ S-CSF-cNs around the cc changed from a predominantly dorsal-ventral localization at the cervical spinal cord level to an almost exclusively lateral one at more rostral levels. Moreover, we showed that the dendritic length of S-CSF-cNs also changed along the medullospinal axis in an orientation-dependent manner. At the level of the cervical spinal cord S-CSF-cNs with a ventral localization had the longest dendrites while in the brainstem S-CSF-cNs with lateral localizations had the longer dendrites. Although we do not have, so far, any data regarding the functional consequences of such an organization, one might suggest that this specific distribution and organization along the cc could lead to specific projection pathways as well as innervations of specific territories. Answering this question might give us insights into the specific function of S-CSF-cNs at different levels of the medullospinal cc.

### PKD2L1 as a Specific Marker for Medullospinal S-CSF-cNs: Functional Implication

PKD2L1 is a member of the polycystin TRP channel subfamily and was first described in the kidney [41], [42] and subsequently in taste buds [16], [43] where it was suggested to play a role in the detection of the sour taste [16], [44]. More recently, the presence of PKD2L1 was reported in medullospinal S-CSF-cNs [12], [16]. Our data confirm that PKD2L1 is a reliable marker for these cells, and we demonstrated here that PKD2L1 is exclusively expressed on the soma and the dendrite of S-CSF-cNs with an apparent strong level of expression on the bud and none on the axon-like process. This localization on a structure in direct contact with the CSF would therefore support a role for PKD2L1 channels as sensors for changes in the composition of CSF and interstitial medium. This particular subcellular localization also supports the idea of a “functional polarization” of S-CSF-cNs. Through PKD2L1 activity, the bud, dendrite and soma might represent sites of integration of signals from bioactive molecules and/or of change in the composition of the CSF and/or the interstitial medium. Due to the cationic nature of the current flowing through PKD2L1 [12], activation of this channel would have a direct effect on the excitability of S-CSF-cNs and the collected information could be relayed through the axon *via* sodium action potentials to distal partners.

### S-CSF-cNs are Ciliated Neurones

Generally, one distinguishes motile from non-motile/primary cilia on the basis of protein composition and organization [45]. Ependymocytes form a single cell layer that constitutes the wall of the CSF cavities and possess motile cilia involved in the motion of the CSF. Primary cilia are present on the soma of most cells, including neurons, and although little is known about their physiological role, they have been proposed to act as chemo- and/or mechanosensors of fluid composition and/or motion [46] and are also thought to play a role in the development of the neural tube [47]. In lower vertebrates and, to some extent, in mammals, cilia in contact with the CSF were reported on the apical side of CSF-cNs [4], [8], [11], [32], [36]. So far, there is no identified function for these cilia but because of their contact with the CSF they were proposed to act as sensors of CSF composition and motion. These studies based on electron microscopic data did not, however demonstrate the nature of these structures.

In the present study, using classical immunohistochemical techniques with antibodies against cytosolic EGFP, we were able to detect some Ac-αTub^+^ cilia-like structures of a few micrometers of length (1–3 µm) emerging from the bud. Nevertheless, when testing for AC3 immunoreactivity, a selective marker of primary cilia [33], we observed numerous AC3^+^ structures in our sections but in S-CSF-cNs AC3^+^ cilia where present only on their somata. This is the first demonstration of a primary cilium on the soma of identified medullospinal S-CSF-cNs, and fits well with one recent study using transmission and scanning electron microscopy in mouse spinal cord. There, cells around the spinal cord cc with a morphology of CSF-cNs exhibited on their soma a single cilium of indeterminate identity [32]. Primary cilia have been suggested to play a role in developmental processes [48] and to have chemoreceptive functions including detection of peptides, hormones or neurotransmitters diffusing from the CSF into the interstitial liquid [46]. One could therefore suggest that due to the primary cilia on their soma, PKD2L1^+^ S-CSF-cNs would be capable of sensing modifications in the motion or composition of interstitial medium. Interestingly, the presence of polycystin-2, a protein of the same family as PKD2L1, in the basal region of the primary cilium has been described in kidney epithelia and vascular endothelial cells and shown to confer to these cells a role in mechanodetection of the flow of primary urine or blood, respectively [49]. Prior studies examining the morphology of the dendritic bud in mouse and rat spinal cord reported that the bud would be round-shaped (3 to 5 µm diameter) and in general devoid of microtubule-based cilia [50], [51]. Our data would need to be confirmed by a dedicated study of the bud ultrastructure, but as suggested by previous reports [8], [11], we did notice thin short Ac-αTub^+^ and AC3 negative cilia-like structures on the PKD2L1^+^ terminal protrusion; a result that might further support its role as a CSF sensor.

### Phenotype and Synaptic Contacts

Numerous studies carried out on the spinal cord of several low vertebrates species [35], [52], [53] as well as mammals [6], [8], [11] have shown that CSF-cNs are GABAergic. Nevertheless, to our knowledge, there is no quantitative and comparative data available on the phenotype of spinal and medullar CSF-cNs. Here using immunohistochemical techniques on adult mouse tissue, we showed that ∼80% of medullospinal S-CSF-cNs were immunoreactive to GABA and GAD67. Further, our results suggested that medullar S-CSF-cN would be capable of synthesizing GABA through GAD67 activity and of releasing it at their axonal terminals. We were not able to determine the phenotype of the remaining ∼20% of PKD2L1^+^ S-CSF-cNs. However, we cannot exclude the possibility that some GABAergic S-CSF-cNs could not be detected due to low levels of GABA/GAD expression. On the other hand, these S-CSF-cNs might represent a neuronal population with a different phenotype as suggested by several studies [7], [9], [10], [54]. In lower vertebrates, CSF-cNs were shown to express dopamine or 5-HT [35], [36] but our results are in agreement with previous studies carried out on mice or rat spinal cord tissues where 5-HT and dopamine expression was not observed in CSF-cNs [9], [10]. Another possibility that we did not test was that the remaining PKD2L1^+^ S-CSF-cNs could express either the aromatic L-amino acid decarboxylase (AADC) to synthesize monoamines [7], [9], [54] or leucine- and methionin-enkephalin [10] as reported for some CSF-cNs in mice.

Complementing these results, we found a strong labeling against GAD67 in the parenchyma and around the cc (fig. 6B). This GAD67^+^ staining appeared punctate, in close apposition with the soma of PKD2L1^+^ S-CSF-cNs and might correspond to GABAergic terminals known to accumulate GAD [55]. This result corresponds to previous electron microscopic data showing that spinal CSF-cNs are in contact with terminals containing small clear vesicles characteristic of GABAergic boutons [6]. We also recently demonstrated at the functional level that medullar S-CSF-cNs receive strong GABAergic synaptic inputs [12]. Taken together these results indicate that PKD2L1^+^ S-CSF-cNs are modulated by functional GABAergic terminals likely arising from local interneurons. We also observed fibers positive for TH, 5-HT and ChAT in close contact with PKD2L1^+^ S-CSF-cNs suggesting some catecholaminergic, serotoninergic or cholinergic modulation of S-CSF-cNs. This is supported on the one hand by the presence of cholinergic neurons in the dorsal motor nucleus of the vagus nerve that could project towards S-CSF-cNs and on the other hand by immunohistochemical studies showing that cells around the cc with the morphology of CSF-cNs were strongly positive for type 6 5-HT receptors [56] (see also www.Gensat.org BAC Address: RP23-65B23). Our results support the idea that S-CSF-cNs are primarily GABAergic cells, receive GABAergic inputs, and might be modulated by other neurotransmitters such as acetylcholine and 5-HT.

### PKD2L1^+^ S-CSF-cNs are in an Intermediate State of Maturity

Most CNS neurons go through a specified maturation process, but in regions such as the olfactory bulb or the dentate gyrus of the hippocampus new neurons are still formed even in adulthood [57], [58]. More recently it was reported that the region surrounding the cc of the spinal cord might represent a new stem cell niche. Stem cells present in this region could be capable *in vivo* of dividing and generating astrocytes and oligodendrocytes while *in vitro* they form neurospheres [24], [25], [31]. In mammals, this region is also suggested to take part in reparatory processes following spinal cord injury [23], [25]. Among the cells found around the cc, some CSF-cNs were shown to express markers associated with neuronal immaturity such as DCX or PSA-NCAM in juvenile [8] and adult rodents [8], [11], [26].

First, we show that in adult animals the vast majority of S-CSF-cNs still expressed DCX (∼90% of cells) as well as HuC/D although in a lower proportion of cells (∼60% of cells). Since these markers are found both in young post-mitotic and immature neurons and in new neurons formed during adult neurogenesis [58], [60] our data suggest that medullospinal PKD2L1^+^ S-CSF-cNs have conserved immature characteristics or are young neurons. Based on several studies indicating that spinal stem cells do not generate new neurons in adults [24], [29] and that CSF-cNs were not the result of postnatal or adult neurogenesis [8], [29], it is unlikely that S-CSF-cNs represent newly formed neurons. Interestingly, recent findings described neurons in the layer II of adult mouse cerebral cortex that were not newly formed yet still expressed DCX and PSA-NCAM, and thus remained in an immature state [61]. In adult mice, we were unable to detect any immunoreactivity in PKD2L1^+^ S-CSF-cNs against PSA-NCAM. This is in contrast to previous reports [8], [11], [31], [34]. A potential explanation for this discrepancy might be the more rostral sections examined in our analysis (cervical spinal cord and brainstem) where little or no PSA-NCAM immunoreactivity was observed [59]. Finally, the lack of mature axonal markers might correspond to this intermediate state of maturity. Neurofilaments become the dominant axonal constituent only as neuronal maturation advances [39], [40]. Therefore, the absence in S-CSF-cNs of immunoreactivity against 160 kDa (this study) and of 200 kDa neurofilaments [29] along with the reported expression of GAP43 in spinal CSF-cNs and their presumed fibers [11] would further support the idea that S-CSF-cNs are in an intermediate state of maturity.

During the embryonic stage, Nkx6.1 homeodomain proteins are strongly expressed in the ventral region of the caudal neural tube and enables the differentiation of ventral progenitors into somatic motoneurons (sMN), ventral (V)2 and V3 neurons [62], [63]. The level of Nkx6.1 expression was shown to progressively decrease during CNS development, to become restricted to the region surrounding the cc of the spinal cord by embryonic day 18 [64] and to remain expressed by some CSF-cNs at adult stages [31]. Here we demonstrated in adult mice first that almost all PKD2L1^+^ S-CSF-cNs strongly expressed Nkx6.1 and second that this high level of Nkx6.1 expression was present in PKD2L1^+^ S-CSF-cNs from the cervical spinal cord up to the rostral part of the brainstem. We observed little expression in other cells around the cc (fig. 7B). These results suggest that PKD2L1^+^ S-CSF-cNs might originate from progenitors common with sMN and V2-3 neurons and reinforce the idea that S-CSF-cNs retain a “non-mature” phenotype even at adult stages. Because of the apparent incomplete maturation state of most PKD2L1^+^ S-CSF-cNs in adult animals, one could suggest that these neurons might have retained maturation and/or migration capabilities that would be activated under circumstances and conditions yet to be determined. One hypothetical trigger for activation of S-CSF-cNs might be tissue injury and the regenerative processes that follow. Indeed, it is well established that under those circumstances, regeneration and tissue repair partially recapitulate morphogenesis by up-regulating, for instance, transcription factors like Pax6 and Olig2 [65]. One could therefore suggest that the selective expression in S-CSF-cNs of Nkx6.1, another transcription factor for neuronal pattern determination, could prompt these cells to participate in post-traumatic processes such as axon growth, rewiring and even neuronal migration.

In young rats, Marichal and colleagues [8] provided evidence that CSF-cNs were negative for NeuN. This result is in agreement with the DCX^+^, PSA-NCAM^+^ and HuC/D^+^ phenotype and confirms the immature state of spinal CSF-cNs. These authors also suggested that cells present in the spinal ependyma, presumably CSF-cNs, were negative for NeuN in tissues obtained from 3 week-old rats (P21). In our study however, we showed that in adult mice (∼P90) S-CSF-cNs did express NeuN along with the immature markers (see also [29]). Our results also suggested that a rostro-caudal gradient of maturation might exist for S-CSF-cNs since the percentage of NeuN^+^/PKD2L1^+^ S-CSF-cNs changes along the rostro-caudal axis. In a sense, these results are unexpected since during development or neurogenesis NeuN expression is supposed to increase with neuronal maturation while DCX expression should decrease and disappear in parallel [58]. However, in the olfactory bulb and the dentate gyrus a temporary overlap between DCX/PSA-NCAM and NeuN expression was reported in newly formed neurons [58]. A similar situation is also observed for the immature neurons in layer II of the cerebral cortex [61]. All in all, the level of NeuN expression is much lower in S-CSF-cNs than in PKD2L1 negative neurons of the same region, indicating once again that S-CSF-cNs exist in an intermediate degree of neuronal maturation (fig. 8B).

## Conclusion

Our study represents the first in-depth analysis of the morphology and distribution of PKD2L1^+^ CSF-cNs in the brainstem of mammals. We show that CSF-cNs correspond to a morphologically homogeneous neuronal population that has a subependymal localization and extends from the cervical spinal cord up to the rostral brainstem. We also demonstrate that S-CSF-cNs exhibit a primary cilium on their soma contacting the interstitial liquid and a protrusion at the end of their dendrite in contact with the CSF. These two appendices might enable S-CSF-cNs to sense both the pericellular fluid and the CSF. We further demonstrate that PKD2L1, selectively expressed on the somatodendritic compartment, represents a specific marker for S-CSF-cNs. Due to modulation of this channel by pH and osmolarity [12], we suggest that PKD2L1 might confer to S-CSF-cNs their ability to detect and integrate changes in the composition of the CSF and the interstitial medium. Our results also indicate that these neurons are mostly GABAergic, are likely contacted by GABAergic inputs [12], and potentially by monoaminergic and cholinergic terminals. Finally, our data indicate that PKD2L1^+^ S-CSF-cNs exist in an intermediate stage of maturity. These neurons display many mature properties: they are polarized cells with an axon-like projection, display a robust action potential discharge activity, are integrated in a neuronal network [12], and express NeuN. On the other hand, NeuN expression is lower in S-CSF-cNs than in surrounding neurons, they do not express mature axonal markers, and they continue to concomitantly express immature markers. Much work remains to characterize the function of these cells and their synaptic targets in the adult CNS. Further investigations will be invaluable for determining first a potential link between S-CSF-cNs and medullospinal stem cells and second if these neurons can be activated under circumstances such as regeneration.
